# Retraction: Innate Immune Activation by Inhaled Lipopolysaccharide, Independent of Oxidative Stress, Exacerbates Silica-Induced Pulmonary Fibrosis in Mice

**DOI:** 10.1371/journal.pone.0155388

**Published:** 2016-05-09

**Authors:** David M. Brass, Jennifer C. Spencer, Zhuowei Li, Erin Potts-Kant, Sarah M. Reilly, Mary K. Dunkel, Joseph D. Latoche, Richard L. Auten, John W. Hollingsworth, Cheryl L. Fattman

The authors of the published article have identified concerns about the reliability of a subset of the reported data, specifically the data underlying Figures 7–9 and the observation that NAC alters the response to silica and LPS in mouse lung. The authors have discovered discrepancies between the raw data from the pulmonary function laboratory and the data it provided for publication, and have concerns about the protocols followed during the animal exposures from which those data were obtained. Figures 1–6 are not affected.

In light of the concerns identified, the authors retract this publication.
